# The Effects of an Initial Extreme Drought and Chronic Change in Precipitation on Plant Biomass Allocation in a Temperate Grassland

**DOI:** 10.1002/ece3.71625

**Published:** 2025-09-10

**Authors:** Amira Fatime Vörös, Andrea Mojzes, Imre Cseresnyés, Tibor Kalapos, Miklós Kertész, Balázs Könnyű, Gábor Ónodi, György Kröel‐Dulay

**Affiliations:** ^1^ HUN‐REN Centre for Ecological Research Institute of Ecology and Botany Vácrátót Hungary; ^2^ Doctoral School of Biology Institute of Biology, ELTE Eötvös Loránd University Budapest Hungary; ^3^ HUN‐REN Centre for Agricultural Research Institute for Soil Sciences Budapest Hungary; ^4^ Department of Plant Systematics, Ecology and Theoretical Biology, Institute of Biology ELTE Eötvös Loránd University Budapest Hungary; ^5^ HUN‐REN Centre for Ecological Research Institute of Evolution Budapest Hungary

**Keywords:** biomass allocation, chronic drought, climate change, drought legacy, extreme event, phytomass

## Abstract

Drying climate may strongly affect plant productivity and standing biomass and, thus, ecosystem carbon cycling. Whilst drought effects on grassland aboveground plant biomass (AGB) have been widely studied and are generally negative, reports on belowground plant biomass (BGB) are much fewer, and results are more variable. In a full factorial experiment in a temperate grassland, we studied the legacy effect of an initial extreme drought, conducted in 2014, and the effect of four levels of chronic precipitation change: water addition, ambient precipitation, moderate drought and severe drought between 2015 and 2018, on AGB, BGB and BGB/AGB in 2019. AGB was negatively affected by chronic drought and also by extreme drought within each level of chronic precipitation change except severe drought. Total BGB was not influenced by extreme drought but was negatively impacted by chronic severe drought. Responses were the strongest in the shallow soil layer (0–10 cm), where both extreme and chronic droughts decreased belowground biomass, and weak in the deep soil layer (10–20 cm). BGB/AGB was not altered by extreme drought but increased in chronic drought plots because the decrease of AGB was much greater than the decrease in BGB. Our results indicate that the legacy of previous extreme drought and chronic change in precipitation are both important in shaping biomass pools and allocation in grasslands, sometimes with interactive effects.

## Graphical abstract

We studied the legacy effect of an initial single extreme drought and the effect of four levels of chronic precipitation change on aboveground plant biomass (AGB), belowground plant biomass (BGB) and BGB/AGB in the 5th year after the start of the experiment. Drought responses were stronger for AGB than those for BGB and in the shallow soil layer (0–10 cm) than in the deep soil layer (10–20 cm). Our results indicate that the legacy of previous extreme drought and chronic change in precipitation are both important in shaping biomass pools and allocation in grasslands, sometimes with interactive effects.

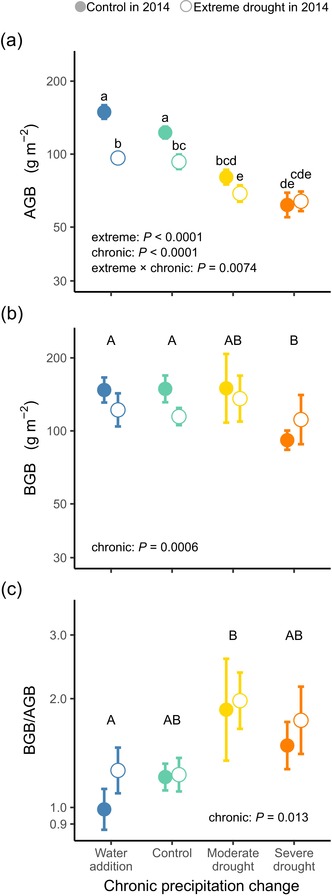

## 1 Introduction

Average global precipitation is projected to increase during the 21st century, but with substantial regional and seasonal differences (Lee et al. 2021). In addition to changing climatic means, climate change is also accompanied by increased frequency and severity of climate irregularities and extremes (Reyer et al. 2013; IPCC 2021). Climate extremes such as heavy precipitation and severe drought can strongly alter regional carbon balance, as these events can cause a substantial reduction in the carbon sink (Reichstein et al. 2013; Frank et al. 2015).

Grasslands (including savannas, open shrublands and tundra) cover a large part of the biosphere (Lieth 1973; Whittaker and Likens 1973; White et al. 2000), account for an estimated 36% of the global terrestrial net primary production (Saugier et al. 2001) and are an important carbon sink (Scurlock and Hall 1998). As the primary productivity of grasslands strongly depends on the amount and timing of precipitation (Sala et al. 2012; Craine 2013), altered precipitation amounts and more frequent extreme precipitation events will likely have large impacts on grassland productivity. Despite the high number of rainfall manipulation experiments and synthesis studies on the topic (Unger and Jongen 2015; Wilcox et al. 2017; Li et al. 2018; Zhang and Xi 2021; Kröel‐Dulay et al. 2022; Wang et al. 2022; Guasconi et al. 2023), the complexity of the responses of grassland productivity to precipitation changes, particularly in the longer term, is not fully understood. One source of variation may arise from the different sensitivities of above‐ and belowground productivity to altered precipitation (Wilcox et al. 2017; Li et al. 2018; Zhang and Xi 2021; Wang et al. 2022; Guasconi et al. 2023).

In general, aboveground plant biomass (AGB) and productivity in grasslands increase with precipitation (Unger and Jongen 2015; Li et al. 2018; Zhang and Xi 2021; Wang et al. 2022), and this relationship holds true both in spatial (between sites) and temporal (within sites) scales (Sala et al. 2012; Knapp et al. 2017). Previous studies consistently showed that the sensitivity of aboveground productivity to increased or decreased precipitation was higher in arid than in mesic sites, and water addition had a greater impact on aboveground productivity than water reduction (positive asymmetry; Unger and Jongen 2015; Knapp et al. 2017; Wilcox et al. 2017; Li et al. 2018). However, extreme precipitation events may reverse this asymmetry, with stronger adverse effects of severe droughts on aboveground net primary productivity (ANPP) than positive impacts of extreme wet periods (Knapp et al. 2017; Wilcox et al. 2017).

Meta‐analyses of precipitation manipulation experiments in grasslands showed that, in contrast to ANPP or AGB, there is high variation in the responses of belowground productivity to precipitation changes. In most studies, belowground plant biomass (BGB) or productivity increased with water addition and decreased in response to drought (Wilcox et al. 2017; Li et al. 2018; Wang et al. 2022; Guasconi et al. 2023), but no change was also reported for both precipitation increase and decrease (Zhang and Xi 2021). Sensitivity of belowground net primary productivity (BNPP) to increased precipitation may be greater (Li et al. 2018), lower (Wang et al. 2022) or similar (Wilcox et al. 2017) to the sensitivity to decreased precipitation. These studies consistently showed that water addition exerted a greater effect on ANPP than on BNPP (Wilcox et al. 2017; Li et al. 2018; Wang et al. 2022). By contrast, when comparing the sensitivity of aboveground and belowground productivity to drought, results are more diverse: reduction in BNPP (or root biomass) may be smaller than that of ANPP (Li et al. 2018; Guasconi et al. 2023) or not significantly different from the magnitude of ANPP response (Wilcox et al. 2017; Wang et al. 2022). Such a high variation highlights the need to improve our understanding of BGB responses to precipitation changes, particularly to drought.

Amongst terrestrial biomes, grasslands have one of the highest root‐to‐shoot biomass ratios (Mokany et al. 2006; Qi et al. 2019), and the highest fraction of total net primary productivity (NPP) allocated belowground (Gherardi and Sala 2020). Thus, a large proportion of plant biomass exists in grassland soils (Ma et al. 2021), and the responses to climate change may strongly affect the global carbon cycle. In these ecosystems, below‐ to aboveground biomass ratio (BGB/AGB) and thus the fraction of NPP occurring in the soil are highly dependent on climate and decrease with increasing mean annual precipitation and temperature across grassland sites (Hui and Jackson 2006; Mokany et al. 2006; Qi et al. 2019; Gherardi and Sala 2020). Consistent with the global patterns of biomass allocation along precipitation gradients, BGB/AGB generally decreased with increased precipitation and increased in response to drought in rainfall manipulation experiments (Zhang and Xi 2021; Wang et al. 2022). Drought may increase biomass allocation to roots to enhance water and nutrient uptake from the soil, whilst water addition may result in increased aboveground growth at the expense of roots to maximise light capture (Tilman 1988). Besides altering biomass allocation between aboveground and belowground plant parts, another important mechanism by which plants can optimise water and nutrient acquisition under different water supplies is changes in root depth distribution in the soil (Zhang et al. 2019; Carroll et al. 2021; Ma et al. 2022; Slette et al. 2023). However, our knowledge of the relative importance of these two mechanisms during droughts, changing biomass partitioning between above‐ and belowground organs or redistributing roots vertically, is limited (Zhang et al. 2019).

Drought can not only exert immediate impacts on current‐year plant productivity or biomass but may also have prolonged post‐drought effects that persist after the drought has subsided, often referred to as drought legacy effects (Müller and Bahn 2022; Vilonen et al. 2022). This may occur if productivity fails to fully recover from drought and remains below the control (negative legacy; Yahdjian and Sala 2006; Slette et al. 2023) or not only recovers but also overcompensates in the post‐drought period (positive legacy; Sun et al. 2022; Luo et al. 2023; Ru et al. 2023). Whilst the aboveground biomass or productivity of grasslands has been frequently reported to recover rapidly following drought (within 1–2 years; Hoover et al. 2014; Mackie et al. 2019; Wilcox et al. 2020; Sun et al. 2022), much less is known about the legacy effect of drought on belowground biomass. However, the limited number of studies have showed that extreme climatic events (high temperature or drought) can have a strong lagged effect on root biomass (Zhou et al. 2012; Slette et al. 2023).

In semiarid regions, ecosystems on sandy soils may be highly sensitive to precipitation changes, partly due to the low soil water‐holding capacity (Yang et al. 2010; Huang et al. 2017). This is the case in perennial sand grasslands in Central Hungary, where both observational (Kovács‐Láng et al. 2005) and experimental studies (Mojzes et al. 2018) reported that drought can induce a major shift in species composition, with a persistent dominance shift in the long run (Orbán et al. 2023) These grasslands are characterised by relatively high BGB and root‐to‐shoot ratio, and root biomass and density are reduced in drier years (Simon and Batanouny 1971; Kovács‐Láng 1974). A decrease in precipitation and longer dry periods in summer are projected for Hungary by the end of the 21st century (Torma et al. 2020), yet it is unclear how changing rainfall patterns can affect above‐ and belowground biomass allocation in these grasslands.

Climate manipulation field experiments provide a valuable tool for studying the ecological impacts of the changing climate, particularly to identify the processes underlying ecosystem responses (De Boeck et al. 2015). The need to incorporate extreme events into climate change experiments was recognised long ago (Jentsch et al. 2007). Yet, experimental data on the combined impacts of extreme drought and multiple levels of chronic precipitation change are limited (Backhaus et al. 2014; Slette et al. 2022, 2023).

The overall objective of our study was to investigate plant biomass pools and allocation in a perennial sand grassland in Hungary in a full factorial climate manipulation experiment that combines an initial extreme drought event and four levels of chronic change in summer precipitation ranging from water addition to severe drought. More specifically, we tested the legacy effect of extreme drought and the effects of 4 years of chronic change in precipitation on (i) aboveground biomass, (ii) total belowground biomass and belowground biomass in the shallow and deep soil layers and (iii) below‐ to aboveground biomass ratio. We hypothesised that (H_1_) extreme drought has legacy effects 5 years after the event. Furthermore, we hypothesised that (H_2_) chronic droughts reduce, whilst water addition increases both AGB and BGB, and with increasing drought severity (H_3_) BGB/AGB increases and (H_4_) within BGB, plants allocate more biomass to the deeper soil layer. We sampled plant biomass allocation in the 5th year (2019) following the extreme drought, because this way the amount of water excluded in the initial extreme drought (2014) and that excluded during 4 years of chronic severe drought (2015–2018) were similar, and the limited size of experimental plots did not allow for destructive yearly soil sampling.

## 2 Materials and Methods

### 2.1 Study Site

The study site is in a perennial sand grassland (*Festucetum vaginatae* “danubiale”), located within the Kiskunság National Park, Central Hungary (46.870° N, 19.422° E; 108 m asl.). The climate is temperate with continental and sub‐Mediterranean influences. The mean annual temperature is 11.1°C, and the mean annual precipitation is 594 mm, measured by a standard meteorological station in the study site (2001–2018). Precipitation distribution is relatively even throughout the year, with a peak in June followed by somewhat drier July and August on average (Kovács‐Láng et al. 2000). Therefore, most of the plant production occurs in the April–June period, but the growing season is considered to last from April to September. The soil type is calcaric arenosol, with high sand content (96.7%/1.5%/1.8% sand/silt/clay respectively) and very low (0.34%) humus content, characterised with low (0.065 cm^3^ cm^−3^) field capacity (Cseresnyés et al. 2020). The vegetation is dominated by two perennial C_3_ bunchgrasses, *Festuca vaginata* W. et K. and *Stipa borysthenica* Klokov. Annuals and perennial forbs appear as subordinate elements, and also mosses and lichens are present in notable amounts amongst grass tussocks (Kovács‐Láng 1974). The vegetation is characterised by a low (< 40%) canopy cover (Kovács‐Láng et al. 2000).

### 2.2 Experimental Design

**FIGURE 1:**
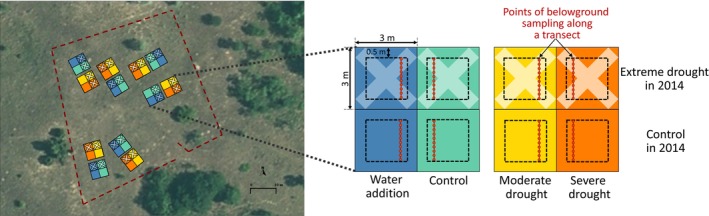
Experimental design of the climate change field manipulation experiment used in this study. The six blocks' spatial arrangement is shown in an aerial photo. There are eight (2 × 4) treatment combinations in six replications, yielding 48 plots in a block design. The plots are 3 × 3 m in size, but only the inner 2 × 2 m areas were used to filter out the edge effect. The soil cores were sampled along a transect (red circles).

The experiment had a full factorial design with two factors: a single extreme drought with two treatment levels in 2014 and a chronic precipitation change treatment with four treatment levels repeated yearly from 2015 (for treatment details see below). This resulted in eight treatment combinations, which were replicated six times in blocks, totaling 48 plots (Figure 1). Plot size was 3 m × 3 m, which included a 0.5 m wide outer buffer zone; all measurements were conducted within the inner 2 m × 2 m area. Although study plots were not hydrologically isolated from each other, the coarse sandy soil of our study site makes lateral water movement unlikely.

To simulate extreme drought, precipitation was completely excluded from extreme drought plots by using transparent polyethylene roofs permanently between 24 April and 18 September 2014. Rain was collected by gutters and diverted away from the plots. The four levels of chronic precipitation treatment, including water addition, ambient precipitation, moderate drought (1 month, from late July to late August) and severe drought (2 months, from late June to late August), were conducted in 2015–2018. Chronic drought treatments were applied by using the same technology as in the extreme drought in 2014. Natural dry periods occur sometimes in July and August; with the chronic drought treatments, we intended to simulate and intensify these events in each year. Water addition was conducted using collected rainwater pumped through sprinklers positioned at 1 m height and in a 1 m × 1 m grid. 25 mm water was added four times: late May, late June, late July and late August, totaling 100 mm each year. During watering, lateral curtains with gutters were used to avoid water entering the adjacent plots. With this design of regular (monthly) watering during the middle part of the growing season, we intended to assure that no major drought events occur. Please note that watering took place also in May 2019 in the watered plots, before biomass sampling in June 2019, but this is unlikely to affect the results, since our study system was generally not responsive to watering (see the Results).

Air temperature and precipitation were measured by permanent sensors (Sensirion SHT75 and Davis DS7852, respectively) at 20 cm height in each plot. Volumetric soil water content (SWC) was measured at 0–30 cm depth by a Campbell CS616 soil moisture sensor. Data were recorded at 10‐min resolution, but we used daily aggregated (summed for precipitation and averaged for other variables) values for further analysis.

### 2.3 Plant Biomass Sampling

#### 2.3.1 Aboveground Plant Biomass

We estimated AGB non‐destructively, an established way of assessing plant biomass in long‐term field experiments (Halbritter et al. 2020). In each plot, we visually estimated the percentage cover of each vascular plant species in four 1‐m^2^ plots in June 2019, the peak of the vegetation period. We converted cover to biomass by species‐specific conversion factors determined through linear regressions between the visually estimated cover and measured AGB in plots outside the experimental area. *R* ^2^ values of the linear regressions were above 0.8 for all species. Previous studies in the same ecosystem found that using visually estimated cover for estimating biomass is at least as good as other non‐destructive estimates, such as NDVI and pin‐point sampling (Ónodi et al. 2017). Conversion factors were obtained for the 13 most abundant species, which accounted for 86% of cover averaged across the 48 experimental plots. For rare species, we used conversion factors of species with the most similar growth forms. AGB included live and standing dead biomass, which was clipped, oven‐dried at 60°C for 48 h and then weighed.

#### 2.3.2 Belowground Plant Biomass

To assess the amount of BGB, we collected soil cores from the experimental plots in June 2019. We took 10 soil cores from each plot systematically along a 2 m transect (Figure 1) from two depths: 0–10 cm and 10–20 cm, as in this vegetation type, the majority of plant roots appear in the 0–20 cm soil layer (Simon and Batanouny 1971). Samples were taken at the exact locations along the transect, at 20 cm intervals, no matter if it was a grass tussock or interspace. For sampling, we used a manual soil auger of 2.7 cm diameter. In each plot, soil core samples were pooled in each depth category, resulting in one sample per depth from each of the 48 plots.

In the laboratory, samples were sieved through a 1 mm mesh size to separate plant parts from sand, then cleaned manually to separate the remaining BGB from other contaminants using forceps. After dry cleaning, samples were soaked in a sieve (1 mm mesh size) with tap water. The cleaned BGB contained not only roots but also rhizomes and shoot bases as well. After cleaning, samples were oven‐dried at 60°C for 48 h and weighed at 0.1 mg accuracy.

### 2.4 Statistical Analysis

All statistical analyses were carried out in R version 4.3.2 (R Core Team 2023). To assess how extreme drought legacy and chronic change in precipitation influenced plant biomass allocation, we applied linear mixed‐effect models using the *nlme* package (Pinheiro et al. 2021). Separate models were used for each of the following response variables: AGB, (total) BGB, BGB/AGB and separately the two belowground layers (BGB 0–10 cm and BGB 10–20 cm). Extreme drought, chronic change in precipitation and their interaction were included as fixed factors, whilst the block was considered as a random factor. To improve normality, response variables were log‐transformed in all cases. The normality and homogeneity of residuals were checked, and to manage heteroscedasticity, we applied *VarIdent* variance structure with explanatory variable chronic change in precipitation (in AGB, BGB/AGB, BGB 0–10 and BGB 10–20 models) or with the explanatory variables interaction (in BGB model). During model selection, we reduced the full models (Table 2a) by removing the non‐significant factors, and the best fitting models were selected by the lowest Akaike index, considering also the model residuals' distribution. We use this reduced model as a final output (Table 2b), but present both models. To detect significant differences between treatments (within groups and where it is needed between groups), we applied post hoc tests with Šidák *p*‐value correction using the *emmeans* package (Lenth 2022). In all analyses, the familywise significance level was *α* = 0.05.

## 3 Results

Extreme drought treatment in 2014 excluded 94% of ambient rainfall (523.5 mm) and decreased average SWC from 5.5% ± 0.1 (mean ± SE, *n* = 24) in control plots to 3.1% ± 0.1 in extreme drought plots during the growing season (1 April–30 September; Table 1). Across the 4‐year chronic precipitation changes (2015–2018), rainfall amounts excluded from severe and moderate drought plots were 42% (138.5 mm) and 21% (70.9 mm) of ambient precipitation during the growing season, respectively, whilst watering treatment increased rainfall by 31% (102.4 mm) compared to control. By the sampling year (2019), which was the fifth year following the extreme drought of 2014, the amount of water excluded in the single extreme drought (2014) and that excluded during 4 years of chronic severe drought (2015–2018) were similar (523.5 mm and 554 mm, respectively). We detected a slightly increased temperature in both the extreme drought plots in 2014 and in the chronic drought plots in 2015–2018 (Table 1).

**TABLE 1:** Growing season (April through September) mean air temperature, sum of precipitation, and average volumetric soil water content in 2014 and the mean between 2015 and 2018, based on daily average micrometeorological data.

	Control in 2014				Extreme drought in 2014			
	Water addition	Control	Moderate drought	Severe drought	Water addition	Control	Moderate drought	Severe drought
2014								
Temperature (°C)	18.2	18.3	18.3	18.4	18.6	18.5	18.6	18.6
Precipitation (mm)	554.1	554.1	554.1	554.1	30.6	30.6	30.6	30.6
Soil water content (%)	5.2	5.4	5.7	5.7	3.1	3.5	3.0	3.0
2015–2018								
Temperature (°C)	19.2	19.2	19.6	19.7	19.2	19.2	19.5	19.7
Precipitation (mm)	436.1	333.7	262.8	195.2	436.1	333.7	262.8	195.2
Soil water content (%)	4.6	4.6	4.2	4.2	4.9	4.9	4.3	3.9

**FIGURE 2:**
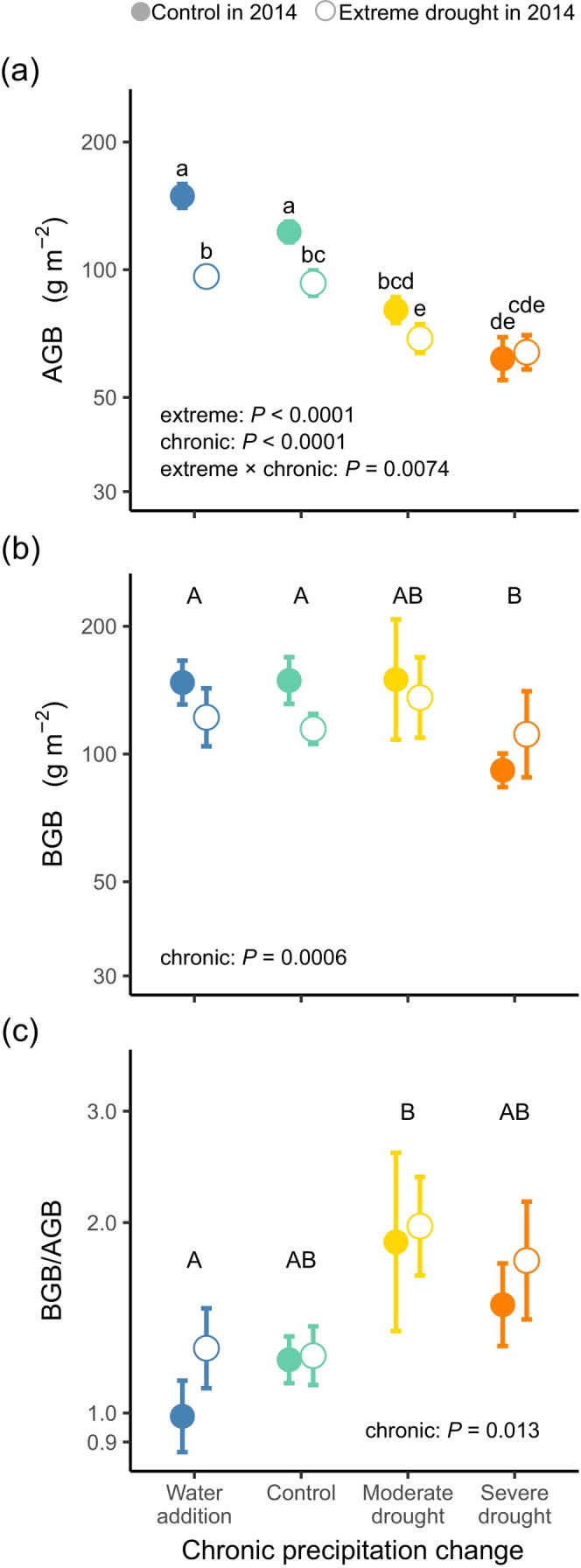
The legacy effect of initial extreme drought and the effect of chronic precipitation change on (a) aboveground plant biomass (ABG), (b) belowground plant biomass (BGB) and (c) below‐ to aboveground plant biomass ratio (BGB/ABG). Values are means ± SE. Lowercase letters indicate significant differences between the treatment combinations, and different capital letters indicate the statistically different groups within chronic precipitation treatment (based on post hoc test with Šidák *p*‐value correction). The familywise significance level was *α* = 0.05; the figures show the results of the final models. Note the log scale used on the y‐axis.

Both extreme drought and chronic change in precipitation affected AGB, with significant interaction between the two variables (Table 2b and Figure 2a). Extreme drought had a negative legacy effect on AGB at all levels of chronic precipitation change except severe drought, where control and extreme drought plots did not differ (Figure 2a). Both moderate and severe drought treatments decreased, whilst water addition did not affect AGB compared to the control.

**TABLE 2:** The legacy effect of the initial extreme drought treatment (conducted in 2014), the effect of the chronic precipitation change treatment, and the treatments interaction on aboveground plant biomass (AGB), belowground plant biomass (BGB), below‐ to aboveground plant biomass ratio (BGB/AGB), and belowground plant biomass in the shallower (0–10 cm) layer (BGB 0–10) and in the deeper (10–20 cm) layer (BGB 10–20) of the soil based on linear mixed effect models.

	Extreme drought in 2014			Chronic precipitation change			Interaction		
	df	*F*	*p*	df	*F*	*p*	df	*F*	*p*
a: Results of full models									
AGB	1	61.5696	< 0.0001	3	68.2298	< 0.0001	3	4.6882	0.0074
BGB	1	1.0829	0.31	3	7.1874	0.0007	3	1.1770	0.33
BGB 0–10	1	5.9922	0.020	3	3.0480	0.041	3	1.6142	0.20
BGB 10–20	1	0.0059	0.94	3	1.4433	0.25	3	0.0707	0.98
BGB/AGB	1	1.6009	0.21	3	3.9607	0.016	3	0.4552	0.72
b: Results after model selection									
AGB	1	61.5696	< 0.0001	3	68.2298	< 0.0001	3	4.6882	0.0074
BGB				3	7.2299	0.0006			
BGB 0–10	1	6.1539	0.018	3	2.6808	0.061			
BGB 10–20									
BGB/AGB				3	4.0594	0.013			

**FIGURE 3:**
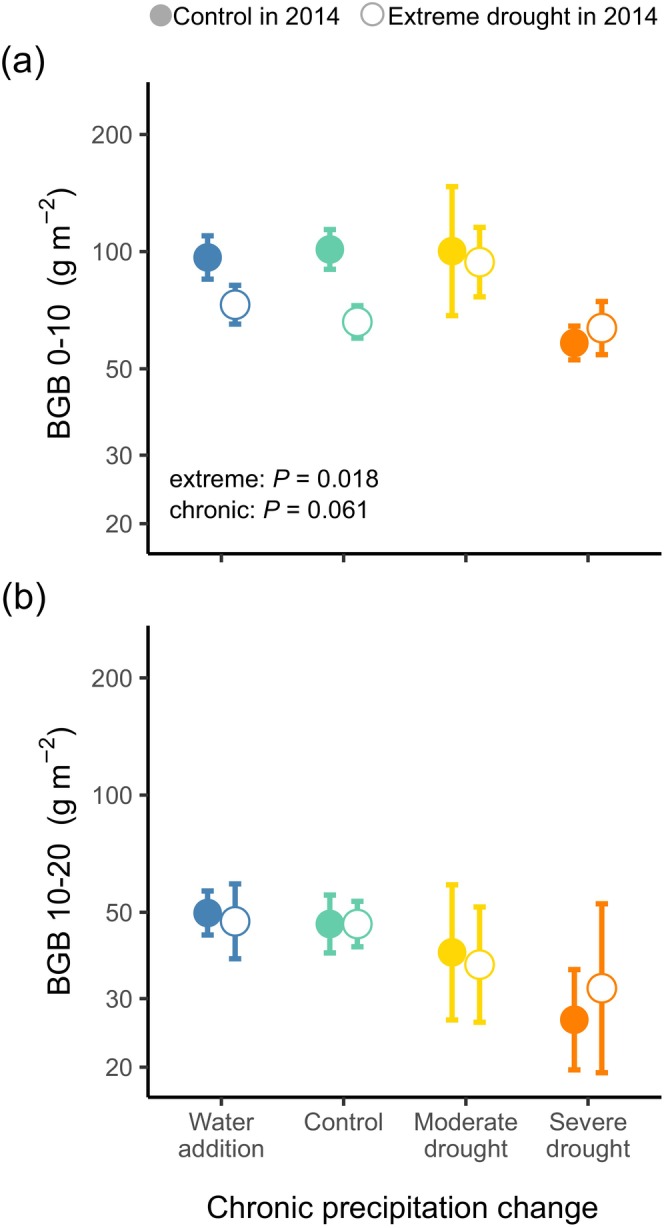
The legacy effect of initial extreme drought and the effect of chronic precipitation change on the amount of belowground plant biomass (a) in the shallower, 0–10 cm layer (BGB 0–10) and (b) in the deeper, 10–20 cm layer (BGB 10–20) of the soil. Values are means ± SE at different treatment combinations; the familywise significance level was *α* = 0.05. Panel (a) shows the result of the final model; in the deeper soil layer (b), there was no significant treatment effect. Note the log scale used on the y‐axis.

We did not see evidence of legacies from extreme drought on BGB, but we did observe the effects of chronic change in precipitation (Table 2b and Figure 2b). In severe drought plots, BGB was lower than in the irrigated and control plots. By contrast, water addition did not influence BGB compared to control (Figure 2b). When looking at the two soil layers separately, BGB in the shallow (0–10 cm) layer was negatively impacted by both legacy effect of initial extreme drought and chronic severe drought (the effect of chronic precipitation change was marginally significant), whilst BGB in the deep (10–20 cm) layer was unaffected by either of the treatments (Figure 3 and Table 2b).

Although there was no legacy effect of extreme drought detected on BGB/AGB, we did observe the effects of chronic changes in precipitation (Table 2b and Figure 2c). There was an increasing trend in BGB/AGB from water addition plots, through control plots, to chronic drought plots, even if post hoc test found significant difference only between water addition and moderate drought plots (Figure 2c).

## 4 Discussion

We aimed to study how plant biomass pools and allocation to aboveground and belowground parts of a semiarid temperate grassland change under experimentally altered precipitation regimes. To our knowledge, our study is the first that examined the legacy effects of extreme drought on both aboveground and BGB under different precipitation regimes (chronic dry, ambient or chronic wet) following an extreme drought event. We found that both AGB and BGB decreased with chronic precipitation reduction, but because the aboveground part responded more strongly, BGB/AGB increased in response to chronic drought. Our results showed that, 5 years after the event, extreme drought still affected AGB and the vertical distribution of BGB, but not the total BGB. We found no influence of water addition on biomass pools and allocation.

### 4.1 Effect of Drought

#### 4.1.1 Aboveground Plant Biomass

Drought legacy effect on aboveground productivity in grasslands can be negative (Yahdjian and Sala 2006; Griffin‐Nolan et al. 2018) or positive (Griffin‐Nolan et al. 2018; Sun et al. 2022; Luo et al. 2023; Ru et al. 2023), but no legacy effect has also been reported (Hoover et al. 2014; Griffin‐Nolan et al. 2018; Mackie et al. 2019; Wilcox et al. 2020). In line with our hypothesis (H_1_), the legacy effect of drought on AGB was detected in our experiment. The lower AGB measured in 2019 in plots exposed to 5‐month rain exclusion in 2014 compared to the respective control plots indicates that extreme drought exerted negative legacy effects on AGB. The drought legacy effect was the largest in plots that were watered in the subsequent years following extreme drought and decreased with the decreasing amount of rainfall that plots received during the recurring precipitation treatments (i.e., control > moderate drought > severe drought). This trend indicates that chronic precipitation changes following extreme drought differentially affected the recovery dynamics of aboveground productivity. Similar to our study, Yahdjian and Sala (2006) also found a greater legacy effect of extreme drought treatment (80% reduction in yearly precipitation) on ANPP in plots that received experimental rainfall addition than in plots that received ambient precipitation in the year following the drought treatment. In our experiment, we observed no evidence of the legacy effect of extreme drought in plots that were exposed to severe drought treatment. This result can be explained by the fact that the 4‐year chronic severe drought alone dropped AGB to a similar level as the single extreme drought event, thus masking the legacy effect of extreme drought. These differences in the magnitude of drought legacy effects between chronic precipitation regimes following extreme drought highlight the importance of background climatic conditions during the process of ecosystem recovery after droughts.

In our study, both moderate (1‐month) and severe (2‐month) recurring droughts substantially decreased AGB, which is in agreement with (H_2_). This result is consistent with the general drought response of aboveground productivity in grasslands reported by many case studies (Yahdjian and Sala 2006; Hoover et al. 2014; Griffin‐Nolan et al. 2018; Mackie et al. 2019; Zhang et al. 2019; Carroll et al. 2021; Ma et al. 2022; Sun et al. 2022; Luo et al. 2023) and meta‐analyses (Li et al. 2018; Zhang and Xi 2021; Kröel‐Dulay et al. 2022; Wang et al. 2022; Guasconi et al. 2023). Moreover, the decline in AGB induced by severe drought treatment was twice as large (48%) as that observed 5 years after extreme drought (24%, both compared to control plots that received ambient rainfall throughout the experiment), even though the total amount of rainfall excluded by the two types of drought treatment was similar (523.5 mm and 554 mm in the extreme drought and chronic severe drought, respectively; see also Methods). However, an important limitation of such a direct comparison is that we measured chronic drought effect in the subsequent year after the drought, whereas we measured extreme drought effect 5 years after the event. Nevertheless, these results point out that not only single extreme drought events but also recurring droughts may have severe impacts on aboveground productivity in grasslands in the near future when the frequency and duration of droughts are projected to increase in several regions of the world (Mirzabaev et al. 2022).

There are several potential mechanisms underlying the concurrent and/or long‐lasting negative effects of drought on AGB at both species and community levels, including plant mortality, decreased tiller or stolon density, reduced reproductive output and changes in the composition of species or functional groups (Müller and Bahn 2022). Previous studies at our study site suggest that a shift in plant species abundances in response to drought may be a possible mechanism behind both the response of AGB to recurring droughts and the legacy effect of the single extreme drought event. Specifically, the 2‐month rain exclusion in the same experiment increased the abundance of a subordinate winter annual grass at the expense of previously dominant perennial grasses (Mojzes et al. 2018). The high sensitivity of perennial grasses to droughts in sand grasslands of the region has also been reported from observational studies (Orbán et al. 2023).

#### 4.1.2 Belowground Plant Biomass

Contrary to AGB, we found no legacy effect of extreme drought treatment on total BGB 5 years after the drought event, which did not support (H_1_). This indicates that BGB was less sensitive to a single extreme drought event or had a greater capacity to recover from drought than AGB. This result contradicts that of Slette et al. (2023), where BNPP was lower in plots that were exposed to extreme drought 4 years earlier than in plots that received ambient precipitation throughout the experiment.

Previous grassland experiments reported variable responses of belowground productivity to recurring droughts, including decrease (Byrne et al. 2013; Carroll et al. 2021; Slette et al. 2023), no change (Byrne et al. 2013; Carroll et al. 2021; Ma et al. 2022) or increase (Liu et al. 2018) in the final year of the drought treatment. In a recent meta‐analysis, Guasconi et al. (2023) found that multi‐year droughts decreased root biomass in humid climates but not in dry climates. In the present study, in agreement with (H_2_), BGB significantly decreased in response to a 4‐year severe drought in a semiarid grassland. A possible explanation for this result is the very low water‐holding capacity of sandy soil at our site (Kovács‐Láng et al. 2000; Várallyay 2005), which may increase the sensitivity of the vegetation to drought. However, we observed a smaller decline in BGB (19%) compared to AGB (41%) in response to severe drought and found no effect of moderate drought on BGB, which suggests that BGB had lower responsiveness to recurring droughts than AGB. Together with previous studies that reported different sensitivity of above‐ and belowground biomass to drought (Li et al. 2018; Carroll et al. 2021; Ma et al. 2022; Guasconi et al. 2023), our results highlight that the responses of both above‐ and belowground biomass should be considered for a reliable estimation of drought‐induced changes in plant biomass.

Analysing the soil layers separately, we found that both the legacy effect of extreme drought and the concurrent effect of 4‐year severe drought treatments were more pronounced in the shallow (0–10 cm) than in the deeper (10–20 cm) soil layer, where there was no evidence of drought effect. In the shallow layer, we found lower BGB in extreme drought plots compared to control plots, which indicates that extreme drought had a multi‐year lagged effect on biomass in the upper 10 cm layer of the soil despite having no influence on total BGB. In addition, the chronic change in precipitation had a marginally significant effect on BGB in the shallow soil layer, which reflected biomass reduction in response to severe drought, as found for total BGB. These responses of BGB in the shallow soil layer to both extreme drought and recurring severe droughts indicate that drought induced a shift in the root system to a deeper layer of the soil to increase plant water uptake, which provided evidence in favour of (H_4_). Previous studies also reported that grasslands allocated proportionally more biomass to the deeper soil layer under drought conditions, resulting from the reduction of BNPP in the upper (0–10 cm) layer of the soil with a parallel increase (Zhang et al. 2019; Ma et al. 2022) or no change (Slette et al. 2023) in BNPP in the deeper (> 10 cm) soil layer. Our results add to the growing evidence that for a better understanding of the drought response of belowground productivity in grasslands, not only changes in total BGB, but also changes in its vertical distribution should be investigated.

#### 4.1.3 Belowground to Aboveground Plant Biomass Ratio

In grasslands, plants generally increase biomass allocation to roots under drought conditions to maximise resource uptake from the soil (Zhang and Xi 2021; Ma et al. 2022; Wang et al. 2022). Despite this general pattern, proportional biomass allocation to roots can also remain unchanged in some grassland sites, even under extreme drought conditions (Zhang et al. 2019; Ma et al. 2022). Our study found no legacy effect of extreme drought on the BGB/AGB ratio 5 years after the drought event. Chronic precipitation changes had an effect—in line with (H_3_)—but a significant difference was observed only between the moderate drought and water addition plots. BGB/AGB tended to increase in response to chronic droughts compared to ambient precipitation (control), resulting from the pronounced decline in AGB and no change (by moderate drought) or a smaller decline (by severe drought) in BGB. These results suggest that in our study system, plants can optimise water and nutrient acquisition under drier conditions not only by redistributing roots vertically along the soil profile but also by changing the biomass allocation between above‐ and belowground plant parts. The drought‐induced change in biomass allocation can result from both species‐level responses and altered community composition (Ma et al. 2022). Unfortunately, species‐level sampling was not feasible in our study, because of the destructive way of sampling belowground biomass, the limited size of our experimental plots and the long‐term nature of the research. Since species reordering occurred in our study site due to drought (Mojzes et al. 2018), the altered species composition may contribute to the observed changes in biomass allocation.

### 4.2 Effect of Water Addition

In contrast with the pronounced effects of chronic droughts on both above‐ and belowground biomass in our experiment, water addition did not affect either AGB, BGB or the BGB/AGB ratio, even though the average amount of water added to watered plots (102.4 mm) was similar to those excluded from drought plots (138.5 mm and 70.9 mm for severe and moderate drought, respectively; see the Methods). These results contradict previous studies reporting that in grasslands, water addition generally increased ANPP and BNPP with a stronger response in aboveground than in belowground production (Wilcox et al. 2017; Li et al. 2018; Wang et al. 2022) and decreased below‐ to aboveground biomass ratio (Zhang and Xi 2021; Wang et al. 2022). The soil in our experimental site is a very coarse‐textured sandy soil, characterised by extremely low water‐holding capacity (Kovács‐Láng et al. 2000; Cseresnyés et al. 2020). We hypothesise that this poor water‐holding capacity may explain why there was no effect of water addition; the surplus water added to the system by irrigation was presumably not retained and thus available for plants. Data on SWC support this explanation: whilst reduced water input led to lower SWC, additional water input did not increase soil moisture (Table 1). In addition, it is also possible that more years of increased water input is needed to result in a detectable effect on biomass allocation. Similar to our results, though, aboveground or both above‐ and belowground production remained unresponsive to increased precipitation in other experiments in semiarid temperate grasslands where water addition only slightly increased SWC (Byrne et al. 2013; Flanagan et al. 2013).

## 5 Conclusions

Our results highlight the importance of studying the effect of altered precipitation regimes and measuring not only AGB but also BGB. AGB was found to be more sensitive to drought than BGB. Because the BGB/AGB often increases in dry conditions, the aboveground response may overestimate the overall biomass‐reducing effect of drought, thus giving inaccurate predictions of the impact of drought on carbon balance in grasslands.

Based on our findings, we conclude that the legacy effects of previous extreme drought events and chronic precipitation changes jointly shape grassland biomass allocation. Our results showed that chronic drought had a generally strong impact on the vegetation's below‐ to aboveground biomass allocation; and even 5 years after the treatment, the effects of extreme drought were also detectable on AGB and the vertical distribution of BGB. Collectively, these results suggest that studies should focus on both of these factors—legacy effects of extreme drought events and chronic precipitation alterations—which change in parallel during climate change.

## Author Contributions

**Amira Fatime Vörös:** conceptualization (equal), data curation (equal), formal analysis (equal), investigation (equal), validation (equal), visualization (equal), writing – original draft (equal). **Andrea Mojzes:** writing – original draft (equal), writing – review and editing (equal). **Imre Cseresnyés:** methodology (equal), writing – review and editing (equal). **Tibor Kalapos:** supervision (equal), writing – review and editing (equal). **Miklós Kertész:** investigation (equal), validation (equal), writing – review and editing (equal). **Balázs Könnyű:** formal analysis (equal), writing – review and editing (equal). **Gábor Ónodi:** data curation (equal), investigation (equal), validation (equal), writing – review and editing (equal). **György Kröel‐Dulay:** conceptualization (equal), funding acquisition (equal), investigation (equal), project administration (equal), supervision (equal), validation (equal), writing – review and editing (equal).

## Conflicts of Interest

The authors declare no conflicts of interest.

## Supporting information


Appendix S1

Appendix S2

Appendix S3

Appendix S4

## Data Availability

The dataset analysed during the current study is available as Appendices S2.
