# Supplementary material for: The Effects of an Initial Extreme Drought and Chronic Change in Precipitation on Plant Biomass Allocation in a Temperate Grassland
Source: Ecol Evol. 2025 Sep 10;15(9):e71625. doi: 10.1002/ece3.71625 (PMC12422408; doi:10.1002/ece3.71625)
Supplement: Supplementary file 1 — Appendix S1 [file ECE3-15-e71625-s001.pdf]

Statistical analysis

Amira Voros

2024-12-11

Contents

1 Data **2**

2 Aboveground biomass **3**

3 Belowground biomass **6**

4 Belowground to aboveground biomass ratio **9**

5 Belowground biomass in 0-10 cm layer **11**

6 Belowground biomass in 10-20 cm layer **14**

1 Data

Response variables:

- *ag* : aboveground biomass
- *bg* : belowground biomass
- *bg.ag* : above- to belowground biomass ratio
- *bg10* : belowground biomass in the shallower soil layer (0-10 cm)
- *bg20* : belowground biomass in the deeper soil layer (10-20 cm)

Factors:

- *drought* : extreme drought treatment in 2014
- *prec* : chronic precipitation change
- *block* : experimental design –> random factor

This file shows the results of the models discussed in the article.

2 Aboveground biomass

```
agb <- lme(logag ~ drought * prec, random = ~1 | block,
    weights = varIdent(form = ~1 | prec), method = "REML",
    data = data1)
```

	numDF	denDF	F-value	p-value
(Intercept)	1	35	6198.922429	0.0000000
drought	1	35	61.569608	0.0000000
prec	3	35	68.229811	0.0000000
drought:prec	3	35	4.688195	0.0074354

```
Linear mixed-effects model fit by REML
  Data: data1
        AIC      BIC   logLik
  -0.6350676 21.32037 13.31753

Random effects:
 Formula: ~1 | block
        (Intercept)   Residual
StdDev:   0.1351666 0.08620446

Variance function:
 Structure: Different standard deviations per stratum
 Formula: ~1 | prec
 Parameter estimates:
        C         L         R         S
1.0000000 0.8551109 1.5730165 3.0150759
Fixed effects:  logag ~ drought * prec
                   Value  Std.Error DF  t-value p-value
(Intercept)     5.005211 0.07816397 35 64.03476  0.0000
droughtX       -0.436613 0.07828930 35 -5.57691  0.0000
precC          -0.194951 0.06559834 35 -2.97189  0.0053
precL          -0.618505 0.06300986 35 -9.81600  0.0000
precS          -0.882780 0.11968180 35 -7.37606  0.0000
droughtX:precC  0.158902 0.09277006 35  1.71286  0.0956
droughtX:precL  0.281149 0.08910939 35  3.15510  0.0033
droughtX:precS  0.471398 0.16925563 35  2.78512  0.0086
 Correlation:
               (Intr) drghtX precC  precL  precS  drgX:C drgX:L
droughtX       -0.501
precC          -0.598  0.597
precL          -0.622  0.621  0.741
precS          -0.328  0.327  0.390  0.406
droughtX:precC  0.423 -0.844 -0.707 -0.524 -0.276
droughtX:precL  0.440 -0.879 -0.524 -0.707 -0.287  0.741
droughtX:precS  0.232 -0.463 -0.276 -0.287 -0.707  0.390  0.406

Standardized Within-Group Residuals:
        Min          Q1         Med          Q3         Max
-1.55045964 -0.53319785 -0.03834292  0.41474232  1.93836234
```

Number of Observations: 48
Number of Groups: 6

contrast	estimate	SE	df	t.ratio	p.value
C R - X R	0.4366	0.0783	35	5.577	0.0001
C R - C C	0.1950	0.0656	35	2.972	0.1388
C R - X C	0.4727	0.0656	35	7.205	<.0001
C R - C L	0.6185	0.0630	35	9.816	<.0001
C R - X L	0.7740	0.0630	35	12.283	<.0001
C R - C S	0.8828	0.1197	35	7.376	<.0001
C R - X S	0.8480	0.1197	35	7.085	<.0001
X R - C C	-0.2417	0.0656	35	-3.684	0.0214
X R - X C	0.0360	0.0656	35	0.550	1.0000
X R - C L	0.1819	0.0630	35	2.887	0.1699
X R - X L	0.3374	0.0630	35	5.354	0.0002
X R - C S	0.4462	0.1197	35	3.728	0.0189
X R - X S	0.4114	0.1197	35	3.437	0.0420
C C - X C	0.2777	0.0498	35	5.580	0.0001
C C - C L	0.4236	0.0463	35	9.147	<.0001
C C - X L	0.5790	0.0463	35	12.504	<.0001
C C - C S	0.6878	0.1118	35	6.153	<.0001
C C - X S	0.6530	0.1118	35	5.842	<.0001
X C - C L	0.1458	0.0463	35	3.150	0.0894
X C - X L	0.3013	0.0463	35	6.507	<.0001
X C - C S	0.4101	0.1118	35	3.669	0.0223
X C - X S	0.3753	0.1118	35	3.357	0.0521
C L - X L	0.1555	0.0426	35	3.653	0.0233
C L - C S	0.2643	0.1103	35	2.396	0.4644
C L - X S	0.2295	0.1103	35	2.081	0.7233
X L - C S	0.1088	0.1103	35	0.987	1.0000
X L - X S	0.0740	0.1103	35	0.671	1.0000
C S - X S	-0.0348	0.1501	35	-0.232	1.0000

Degrees-of-freedom method: containment
P value adjustment: sidak method for 28 tests

drought	prec	emmean	SE	df	lower.CL	upper.CL	.group
C	S	4.12	0.1196	5	3.58	4.66	ab
X	S	4.16	0.1196	5	3.62	4.70	abc
X	L	4.23	0.0629	5	3.95	4.51	a
C	L	4.39	0.0629	5	4.10	4.67	bcd
X	C	4.53	0.0654	5	4.24	4.83	cd
X	R	4.57	0.0782	5	4.22	4.92	d
C	C	4.81	0.0654	5	4.52	5.10	e
C	R	5.01	0.0782	5	4.65	5.36	e

Degrees-of-freedom method: containment
Confidence level used: 0.95
Conf-level adjustment: sidak method for 8 estimates
P value adjustment: sidak method for 28 tests
significance level used: alpha = 0.05
NOTE: If two or more means share the same grouping symbol,
then we cannot show them to be different.
But we also did not show them to be the same.

```
model: logag ~ drought * prec

 drought*prec effect
        prec
drought        R        C        L        S
      C 5.005211 4.810260 4.386706 4.122431
      X 4.568599 4.532549 4.231243 4.157216
```

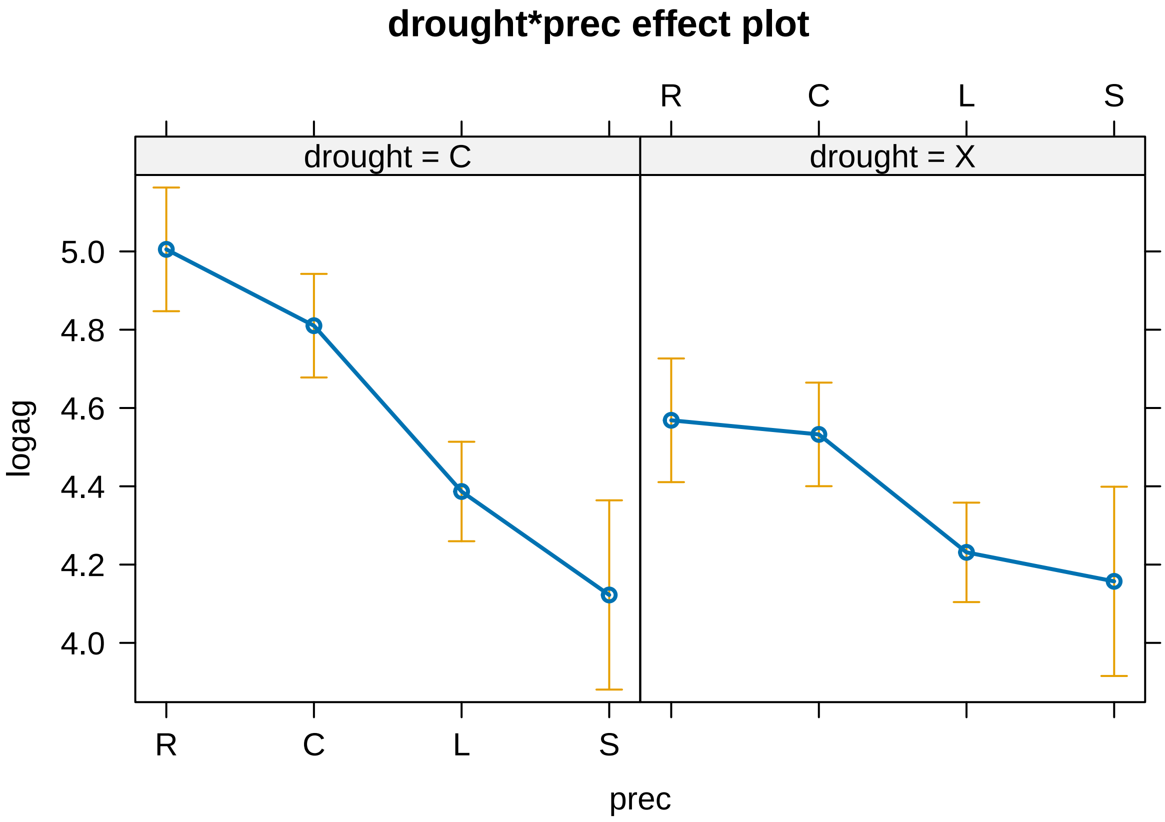

3 Belowground biomass

```
bgb <- lme(logbg ~ prec, random = ~1 | block, weights = varIdent(form = ~1 |
    drought * prec), method = "REML", data = data1)
```

	numDF	denDF	F-value	p-value
(Intercept)	1	39	3346.646344	0.0000000
prec	3	39	7.229885	0.0005692

```
Linear mixed-effects model fit by REML
  Data: data1
       AIC      BIC    logLik
  69.56066 92.75513 -21.78033

Random effects:
 Formula: ~1 | block
        (Intercept)  Residual
StdDev:   0.1781177 0.3749089

Variance function:
 Structure: Different standard deviations per stratum
 Formula: ~1 | drought * prec
 Parameter estimates:
      C*C       C*L       C*R       C*S       X*C       X*L       X*R       X*S
1.0000000 2.0757000 0.6400502 0.3751544 0.5555716 1.0718614 0.7706425 1.2444986
Fixed effects:  logbg ~ prec
                Value  Std.Error DF  t-value p-value
(Intercept)  4.916049 0.10472313 39 46.94330  0.0000
precC       -0.112787 0.10585157 39 -1.06552  0.2932
precL        0.015959 0.16409558 39  0.09725  0.9230
precS       -0.382960 0.09328261 39 -4.10537  0.0002
 Correlation:
      (Intr) precC  precL
precC -0.512
precL -0.330  0.327
precS -0.581  0.575  0.371

Standardized Within-Group Residuals:
        Min          Q1         Med          Q3         Max
-1.49426226 -0.68487713 -0.08742207  0.82659935  1.93274398

Number of Observations: 48
Number of Groups: 6
```

```
 contrast estimate     SE df t.ratio p.value
 R - C       0.113 0.1059 39   1.066  0.8753
 R - L      -0.016 0.1641 39  -0.097  1.0000
 R - S       0.383 0.0933 39   4.105  0.0012
 C - L      -0.129 0.1636 39  -0.787  0.9679
 C - S       0.270 0.0925 39   2.922  0.0340
 L - S       0.399 0.1558 39   2.561  0.0835
```

```
Degrees-of-freedom method: containment
P value adjustment: sidak method for 6 tests

 prec emmean     SE df lower.CL upper.CL .group
 S      4.53 0.0912  5     4.19     4.88  A
 C      4.80 0.1040  5     4.41     5.20   B
 R      4.92 0.1047  5     4.52     5.31   B
 L      4.93 0.1629  5     4.31     5.55  AB

Degrees-of-freedom method: containment
Confidence level used: 0.95
Conf-level adjustment: sidak method for 4 estimates
P value adjustment: sidak method for 6 tests
significance level used: alpha = 0.05
NOTE: If two or more means share the same grouping symbol,
      then we cannot show them to be different.
      But we also did not show them to be the same.
```

contrast	estimate	SE	df	t.ratio	p.value
R - C	0.1127869	0.1058516	39	1.0655192	0.7122164
R - L	-0.0159590	0.1640956	39	-0.0972541	0.9996646
R - S	0.3829595	0.0932826	39	4.1053685	0.0011027
C - L	-0.1287458	0.1636256	39	-0.7868318	0.8599750
C - S	0.2701726	0.0924534	39	2.9222568	0.0282294
L - S	0.3989185	0.1557897	39	2.5606212	0.0661230

```
 model: logbg ~ prec

 prec effect
prec
       R        C        L        S
4.916049 4.803262 4.932008 4.533090
```

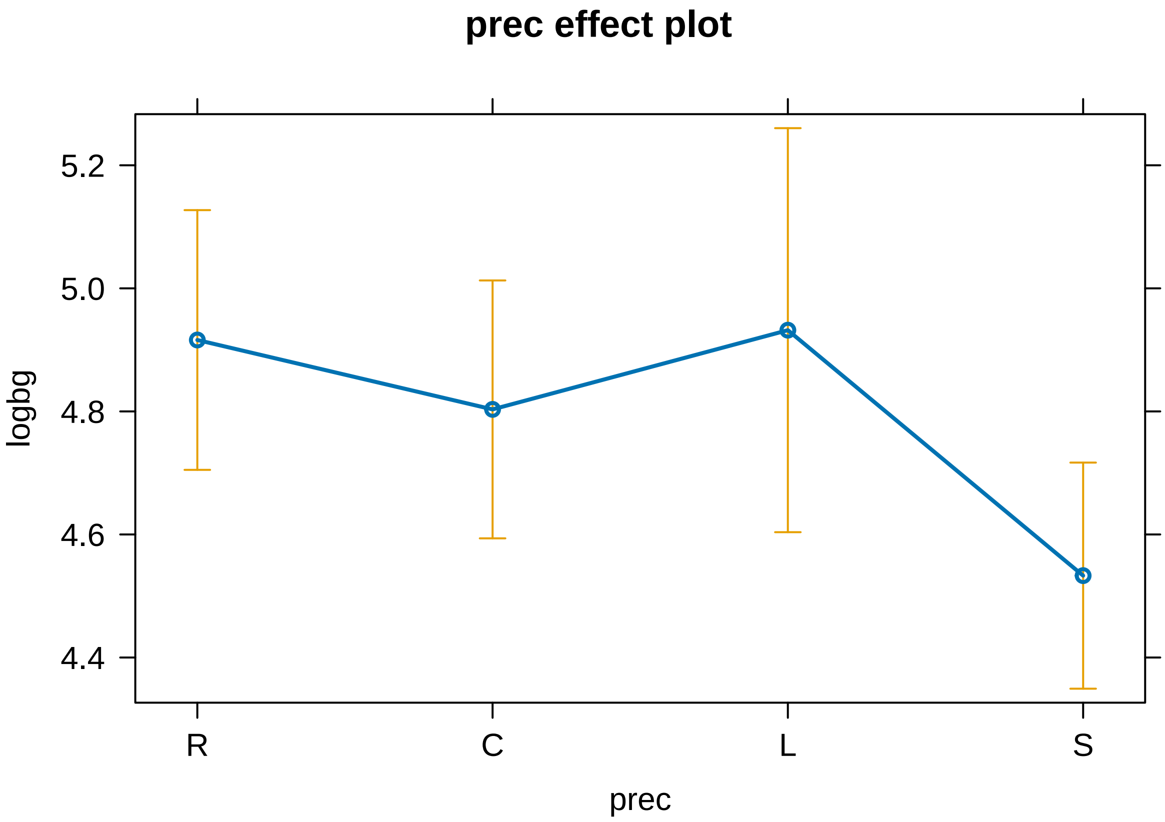
prec effect plot
logbg
5.2
5.0
4.8
4.6
4.4
R
C
L
S
prec

4 Belowground to aboveground biomass ratio

```
bgb.agb <- lme(logbg.ag ~ prec, random = ~1 | block,
    weights = varIdent(form = ~1 | prec), method = "REML",
    data = data1)
```

	numDF	denDF	F-value	p-value
(Intercept)	1	39	12.083481	0.0012635
prec	3	39	4.059428	0.0132806

```
Linear mixed-effects model fit by REML
  Data: data1
      AIC      BIC    logLik
  65.8297 81.88741 -23.91485

Random effects:
 Formula: ~1 | block
        (Intercept)  Residual
StdDev:   0.1339644 0.2131702

Variance function:
 Structure: Different standard deviations per stratum
 Formula: ~1 | prec
 Parameter estimates:
       C        L        R        S
1.000000 2.819579 1.387161 1.926987
Fixed effects:  logbg.ag ~ prec
                Value Std.Error DF   t-value p-value
(Intercept) 0.1118258 0.1013789 39 1.1030480  0.2768
precC       0.0894533 0.1052302 39 0.8500719  0.4005
precL       0.5395727 0.1933694 39 2.7903731  0.0081
precS       0.3629101 0.1461097 39 2.4838195  0.0174
 Correlation:
      (Intr) precC  precL
precC -0.683
precL -0.372  0.358
precS -0.492  0.474  0.258

Standardized Within-Group Residuals:
        Min          Q1         Med          Q3         Max
-1.77614236 -0.66843899 -0.03025574  0.67527554  2.27040731

Number of Observations: 48
Number of Groups: 6

 contrast estimate    SE df t.ratio p.value
 R - C     -0.0895 0.105 39  -0.850  0.9536
 R - L     -0.5396 0.193 39  -2.790  0.0477
 R - S     -0.3629 0.146 39  -2.484  0.1000
 C - L     -0.4501 0.184 39  -2.445  0.1093
 C - S     -0.2735 0.134 39  -2.047  0.2530
 L - S      0.1767 0.210 39   0.841  0.9559
```

```
Degrees-of-freedom method: containment
P value adjustment: sidak method for 6 tests

 prec emmean     SE df lower.CL upper.CL .group
 R     0.112 0.1014  5  -0.2725    0.496  A
 C     0.201 0.0823  5  -0.1109    0.513  AB
 S     0.475 0.1306  5  -0.0204    0.970  AB
 L     0.651 0.1819  5  -0.0383    1.341   B

Degrees-of-freedom method: containment
Confidence level used: 0.95
Conf-level adjustment: sidak method for 4 estimates
P value adjustment: sidak method for 6 tests
significance level used: alpha = 0.05
NOTE: If two or more means share the same grouping symbol,
      then we cannot show them to be different.
      But we also did not show them to be the same.

 model: logbg.ag ~ prec

 prec effect
prec
        R         C         L         S
0.1118258 0.2012791 0.6513985 0.4747359
```

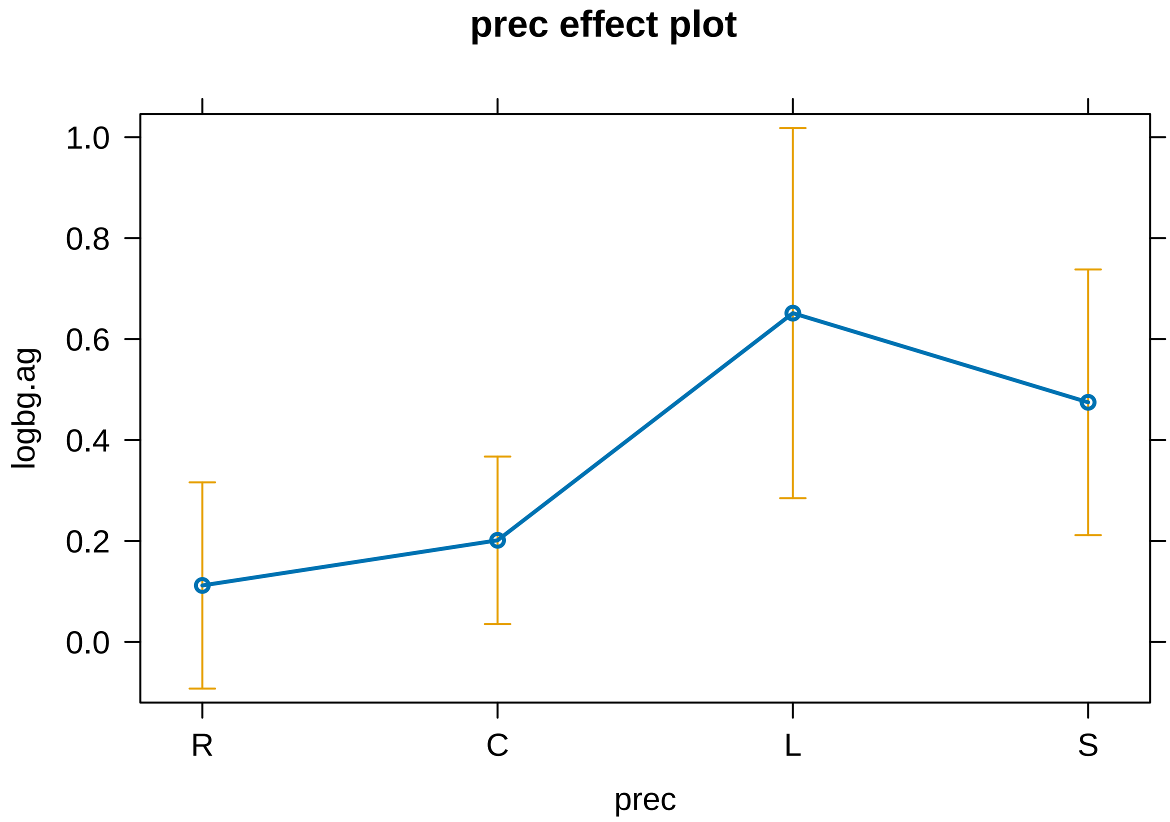

5 Belowground biomass in 0-10 cm layer

```
bgb.10 <- lme(logbg10 ~ prec + drought, random = ~1 |
    block, weights = varIdent(form = ~1 | prec), method = "REML",
    data = data1)
```

	numDF	denDF	F-value	p-value
(Intercept)	1	38	7981.073165	0.0000000
prec	3	38	2.680766	0.0604953
drought	1	38	6.153897	0.0176556

```
Linear mixed-effects model fit by REML
  Data: data1
       AIC      BIC    logLik
  71.03599 88.64799 -25.51799

Random effects:
 Formula: ~1 | block
         (Intercept)  Residual
StdDev: 1.255101e-05 0.2741022

Variance function:
 Structure: Different standard deviations per stratum
 Formula: ~1 | prec
 Parameter estimates:
       C        L        R        S
1.000000 2.638737 1.055109 1.300468
Fixed effects:  logbg10 ~ prec + drought
                Value  Std.Error DF  t-value p-value
(Intercept)  4.551974 0.09668415 38 47.08087  0.0000
precC       -0.027208 0.11502647 38 -0.23654  0.8143
precL        0.144642 0.22486667 38  0.64323  0.5239
precS       -0.322047 0.13250964 38 -2.43037  0.0199
droughtX    -0.241929 0.09752412 38 -2.48070  0.0177
 Correlation:
         (Intr) precC  precL  precS
precC    -0.627
precL    -0.321  0.269
precS    -0.544  0.457  0.234
droughtX -0.504  0.000  0.000  0.000

Standardized Within-Group Residuals:
        Min          Q1         Med          Q3         Max
-1.42331500 -0.72163465 -0.02772913  0.61559651  2.29317508

Number of Observations: 48
Number of Groups: 6

 contrast estimate    SE df t.ratio p.value
 R - C      0.0272 0.115 38   0.237  1.0000
 R - L     -0.1446 0.225 38  -0.643  0.9884
 R - S      0.3220 0.133 38   2.430  0.1137
```

```
 C - L     -0.1718 0.223 38  -0.770  0.9712
 C - S      0.2948 0.130 38   2.271  0.1612
 L - S      0.4667 0.233 38   2.005  0.2748

Results are averaged over the levels of: drought
Degrees-of-freedom method: containment
P value adjustment: sidak method for 6 tests

 prec emmean     SE df lower.CL upper.CL .group
 S      4.11 0.1029  5     3.72     4.50  A
 C      4.40 0.0791  5     4.10     4.70  A
 R      4.43 0.0835  5     4.11     4.75  A
 L      4.58 0.2088  5     3.78     5.37  A

Results are averaged over the levels of: drought
Degrees-of-freedom method: containment
Confidence level used: 0.95
Conf-level adjustment: sidak method for 4 estimates
P value adjustment: sidak method for 6 tests
significance level used: alpha = 0.05
NOTE: If two or more means share the same grouping symbol,
      then we cannot show them to be different.
      But we also did not show them to be the same.
```

contrast	estimate	SE	df	t.ratio	p.value
R - S	0.3220470	0.1325096	38	2.430366	0.0534949
C - S	0.2948387	0.1298065	38	2.271372	0.0762251
L - S	0.4666885	0.2327738	38	2.004901	0.1326660

```
 contrast estimate     SE df t.ratio p.value
 C - X       0.242 0.0975 38   2.481  0.0177

Results are averaged over the levels of: prec
Degrees-of-freedom method: containment

 drought emmean     SE df lower.CL upper.CL .group
 X         4.26 0.0812  5     4.00     4.51  a
 C         4.50 0.0812  5     4.24     4.76   b

Results are averaged over the levels of: prec
Degrees-of-freedom method: containment
Confidence level used: 0.95
Conf-level adjustment: sidak method for 2 estimates
significance level used: alpha = 0.05
NOTE: If two or more means share the same grouping symbol,
      then we cannot show them to be different.
      But we also did not show them to be the same.

 drought emmean     SE df lower.CL upper.CL .group
 X         4.26 0.0812  5     4.00     4.51  a
 C         4.50 0.0812  5     4.24     4.76   b

Results are averaged over the levels of: prec
Degrees-of-freedom method: containment
Confidence level used: 0.95
```

```
Conf-level adjustment: sidak method for 2 estimates
significance level used: alpha = 0.05
NOTE: If two or more means share the same grouping symbol,
      then we cannot show them to be different.
      But we also did not show them to be the same.
```

```
 model: logbg10 ~ prec + drought

 prec effect
prec
       R        C        L        S 
4.431010 4.403802 4.575651 4.108963 

 drought effect
drought
       C        X 
4.500821 4.258892 
```

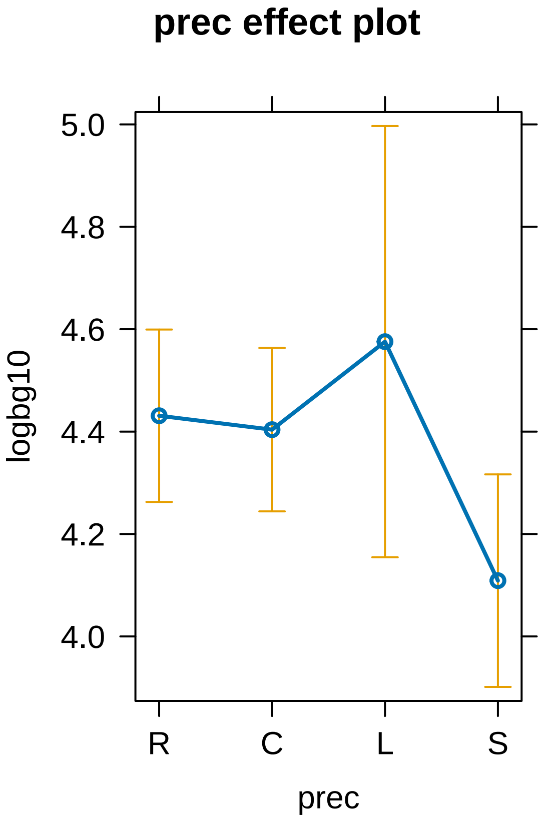

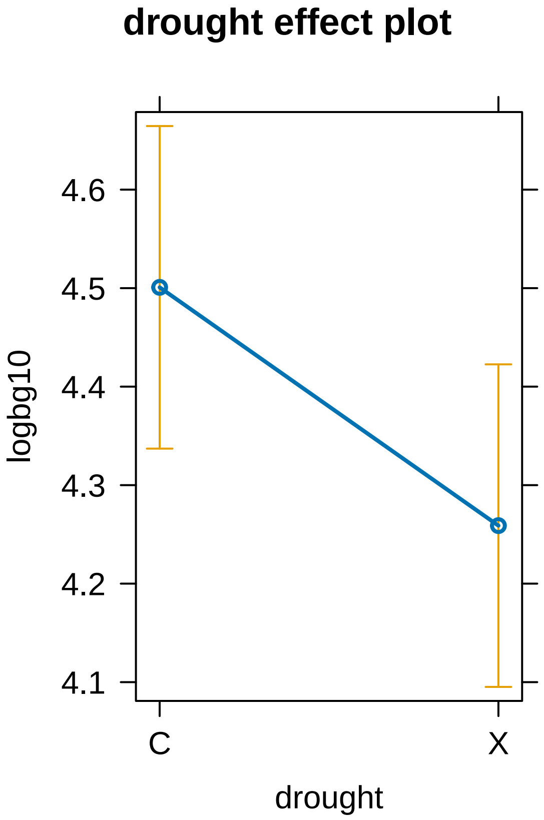

6 Belowground biomass in 10-20 cm layer

```r
bgb.20 <- lme(logbg20 ~ drought * prec, random = ~1 |
    block, weights = varIdent(form = ~1 | prec), method = "REML",
    data = data1)
```

	numDF	denDF	F-value	p-value
(Intercept)	1	35	771.8751339	0.0000000
drought	1	35	0.0059323	0.9390450
prec	3	35	1.4432599	0.2468146
drought:prec	3	35	0.0707026	0.9752233

```
Linear mixed-effects model fit by REML
  Data: data1
       AIC      BIC    logLik
  106.2282 128.1836 -40.1141

Random effects:
 Formula: ~1 | block
        (Intercept)  Residual
StdDev:   0.3113527 0.2253609

Variance function:
 Structure: Different standard deviations per stratum
 Formula: ~1 | prec
 Parameter estimates:
       C        L        R        S
1.000000 3.578018 1.609460 3.847756
Fixed effects:  logbg20 ~ drought * prec
                   Value Std.Error DF   t-value p-value
(Intercept)     3.907605 0.1951489 35 20.023705  0.0000
droughtX       -0.049289 0.2094104 35 -0.235368  0.8153
precC          -0.064859 0.1743300 35 -0.372048  0.7121
precL          -0.232945 0.3609596 35 -0.645348  0.5229
precS          -0.631371 0.3837272 35 -1.645363  0.1088
droughtX:precC  0.048978 0.2465398 35  0.198662  0.8437
droughtX:precL -0.023033 0.5104740 35 -0.045120  0.9643
droughtX:precS  0.235607 0.5426722 35  0.434161  0.6668
 Correlation:
               (Intr) drghtX precC  precL  precS  drgX:C drgX:L
droughtX       -0.537
precC          -0.645  0.601
precL          -0.311  0.290  0.348
precS          -0.293  0.273  0.328  0.158
droughtX:precC  0.456 -0.849 -0.707 -0.246 -0.232
droughtX:precL  0.220 -0.410 -0.246 -0.707 -0.112  0.348
droughtX:precS  0.207 -0.386 -0.232 -0.112 -0.707  0.328  0.158

Standardized Within-Group Residuals:
        Min          Q1         Med          Q3         Max
-1.84360627 -0.57102747 -0.06759169  0.52647131  1.51612656
```

```
Number of Observations: 48
Number of Groups: 6
```
